# Can betamethasone prevent Nicolau syndrome when coadministered with penicillin? A case report

**DOI:** 10.1002/ccr3.5187

**Published:** 2021-12-07

**Authors:** Paria Mojarrad, Behnaz Barikbin, Mohammad Bagher Oghazian

**Affiliations:** ^1^ Clinical Research Development Unit Imam Hasan Hospital North Khorasan University of Medical Sciences Bojnurd Iran; ^2^ Department of Internal Medicine Faculty of Medicine North Khorasan University of Medical Sciences Bojnurd Iran

**Keywords:** benzathine penicillin, betamethasone, coadministration, Nicolau syndrome, protective agents

## Abstract

We present a 33‐year‐old female patient with Nicolau syndrome (NS) who received one injection of benzathine penicillin and one injection of betamethasone to the right buttock, and one injection of benzathine penicillin to the left. NS was seen only in the left buttock, where it was intramuscularly injected with penicillin benzathine alone.

## Graphical abstract

The injection site four days after the injection.

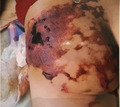

## 1 INTRODUCTION

Nicolau syndrome (NS), also known as livedo‐like dermatitis (LLD) or embolia cutis medicamentosa (ECM), is a rare but severe necrotizing skin complication that often occurs following the intramuscular injection of various drugs. This complication is especially reported with diclofenac and penicillin derivatives. Although NS is more common with intramuscular (IM) injections, several cases of this complication have been reported after subcutaneous, intravascular, and intra‐articular injections.^1^, ^2^ NS usually presents with immediate intense pain after injection, which is often followed by erythematosus ecchymosis, paresthesia, and livedoid. Eventually, NS can lead to various degrees of necrosis extending to the surrounding organs, especially the extremities of the hands, legs, and fingers.^2^ There are no specific diagnostic criteria for NS, however, considering the clinical signs and taking an accurate history of injection help with the diagnosis.^3^ The possible mechanism of this complication seems to be immune system reactions, vasospasm, thrombosis, and embolism in inadvertent intra‐arterial and para‐arterial injections.^2^, ^4^

The management of NS in the initial phase includes symptomatic therapy. With the expansion of symptoms in subsequent phases, antibiotics, corticosteroids, anticoagulants, vasoactive agents, analgesics, and surgical procedures such as debridement, fasciotomy, and skin grafts should be adopted for patients.^3^

In this case report, we present a case of NS in a 33‐year‐old woman after intramuscular administration of simultaneous injection of benzathine penicillin and betamethasone for pharyngitis.

## 2 PATIENT AND OBSERVATION

A 33‐year‐old woman (body mass index (BMI) = 28.51 kg/m^2^) attended the clinic for sore throat. After the diagnosis of pharyngitis was made, two injections of benzathine penicillin 6.3.3. (600,000 unit/vial penicillin‐G benzathine + 300,000 unit/vial penicillin‐G potassium + 300,000 unit/vial penicillin‐G procaine) and one injection of betamethasone were prescribed. The patient had no history of drug allergy, and she had received penicillin in less than a month.

**FIGURE 1:**
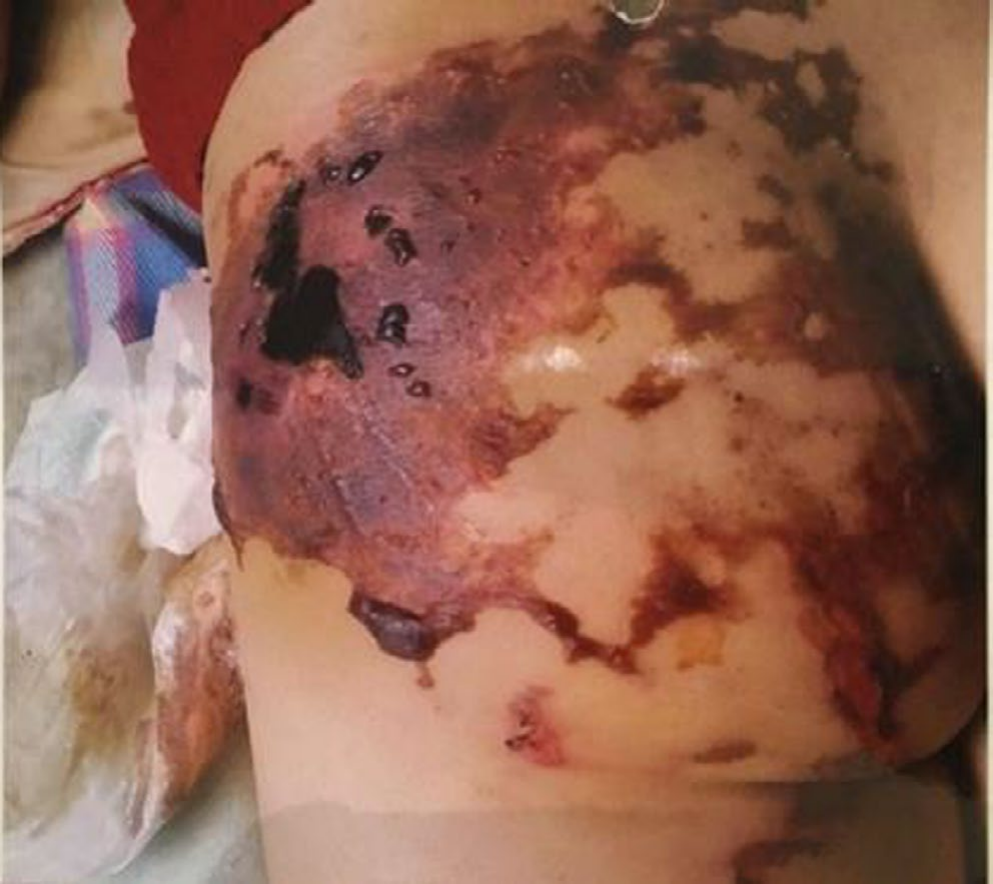
Injection site 4 days after the injection

First, benzathine penicillin and betamethasone were injected concurrently into the right buttock, and the patient had no complaints. Then, the other benzathine penicillin injection was administered to the left buttock. Following the second injection to the left buttock, she experienced intense pain that extended to the legs on the same day. After a few minutes, she experienced paresthesia in both hands and progressive numbness in both legs. Notably, this complication was seen only in the left buttock, where it was intramuscularly injected with penicillin benzathine alone and did not occur in the right buttock coadministered with betamethasone. A warm compressor was applied to relieve the pain in the injection site. On orthopedic examination, the sensory functions and motor abilities were normal. The nurse ensured the proper disinfection and location of the injection site and aspiration of the syringe before injection. After the sensory‐motor system was confirmed to be normal, ibuprofen (400 mg every 8 hours) was prescribed, and the patient was discharged with personal consent. After four days, she returned to the clinic with very severe and prolonged pain and extensive discoloration of the area, along with blisters in the injection site (Figure 1).

Accordingly, she underwent magnetic resonance imaging (MRI) and color Doppler sonography because the lumbosacral region was normal. However, the echogenicity of subcutaneous fat was elevated, which favors inflammation (cellulite). No evidence for collection or hematoma was reported.

**FIGURE 2:**
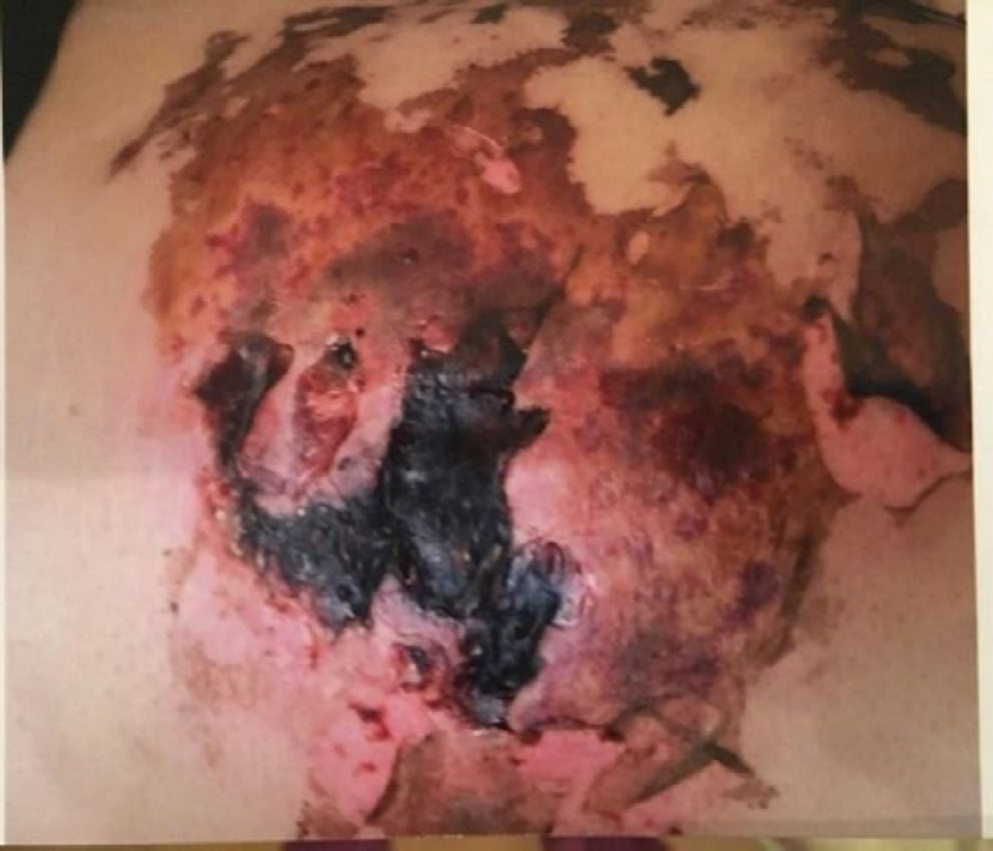
Injection site 23 days after the injection

Twenty‐three days after the injection, the patient's presentations were as follows: intense swelling and reticular bruising of the left buttock, localized infection followed by bursting of the localized blisters, and persistent severe pain (Figure 2).

**FIGURE 3:**
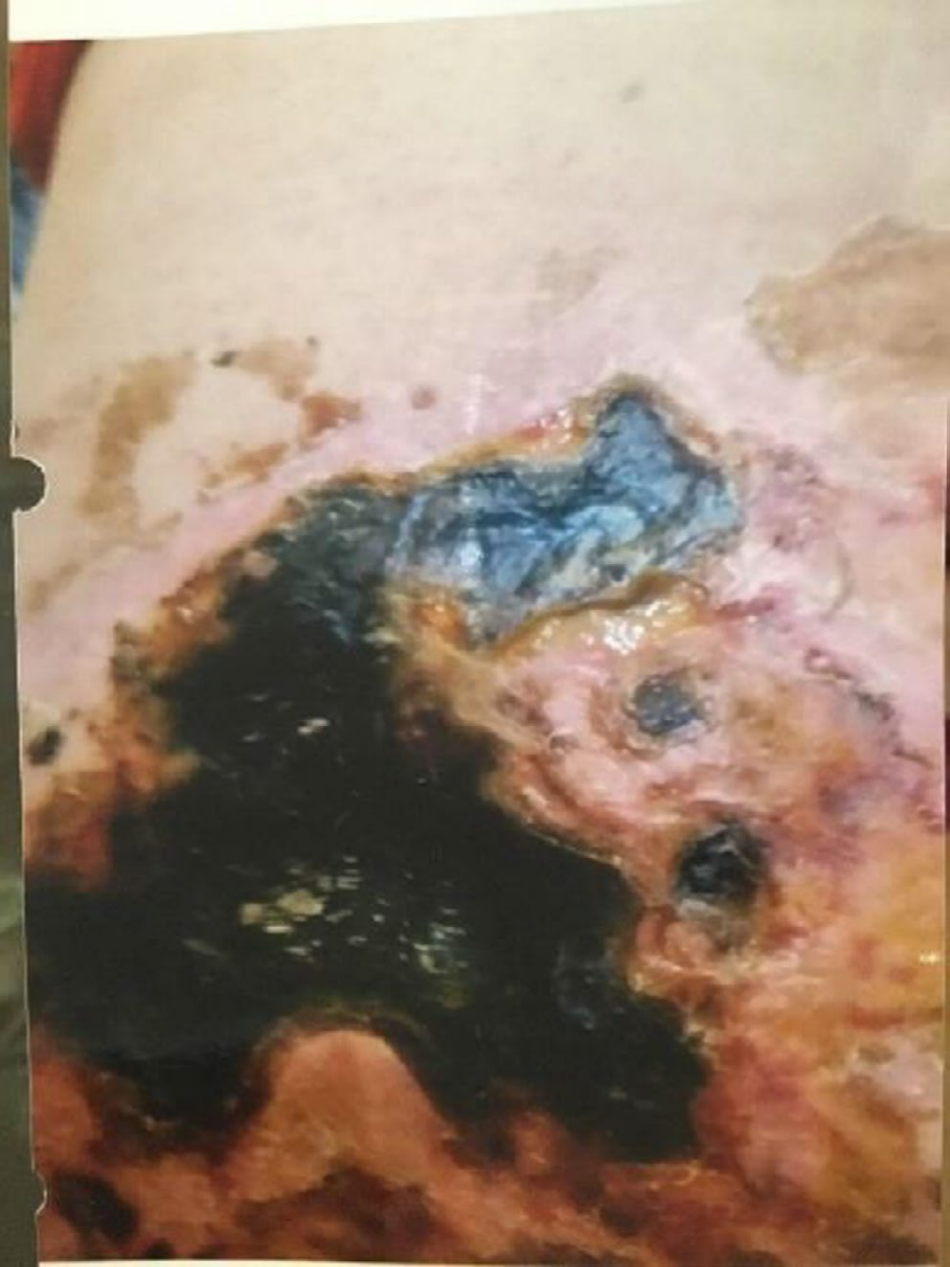
Injection site 42 days after injection

Topical Elase^®^ (Pfizer), zinc oxide, silver sulfadiazine, and oral cephalexin were prescribed to manage the complications. The patient did not have a fever during this period. Improvement was observed in the patient's clinical condition, but the injection site gradually progressed to necrosis (Figure 3).

Forty‐two days after the injection, the patient had normal vital signs and underwent general anesthesia to debride the necrotic tissue with a surface of 3 × 4 cm^2^ and a depth of approximately 1.5 cm. Three days later, debridement was reperformed. Sonography and MRI indicated no damage to the femoral and sciatic nerves and normal blood flow to the femoral vein and obturator artery.

Once more, for the third time, the patient underwent debridement under general anesthesia on day 48 after the initial drug injection. Vital signs, biochemical tests, complete blood count, and electrolytes were normal. Topical hydrocortisone, oral acetaminophen, and cephalexin were prescribed, and the patient was discharged on day 49.

## 3 DISCUSSION

Although Nicolau syndrome is an extremely rare complication, it can be very catastrophic following parenteral drug injection.^5^ Unfortunately, there is no specific test for the diagnosis of this complication, and its etiology is unknown. However, ischemia caused by secondary vasospasm following needle prick, embolism of the injection contents, or pressure on the surrounding vessels may be involved in its pathogenesis.^6^ Paying attention to the appropriate injection site, route of administration, type of prescribed medication, drug preparation, and needle gauge could help prevent Nicolau syndrome. In the case of reconstitution of vials containing powder such as penicillin, the vial contents must be uniform after dilution. It is difficult to ensure that the injection is intramuscular because of the large amounts of adipose tissue, especially in the abdomen and buttocks of most overweight people. Therefore, the BMI of patients is an important contributor to the occurrence of complications, and choosing the right size needle and injection site is crucial.^7^ Accordingly, our case had some risk factors. She was overweight with a BMI of 28.51 kg/m^2^, female sex and age range; as NS has been mostly reported in females and in the age range of 30–40 years old (4).

Since there are no available laboratory tests or specific criteria for NS, before making a definitive diagnosis of NS, all other differential diagnoses should be ruled out. Topical reaction to the drug, compartment syndrome, necrotizing fasciitis, vasculitis, fat embolism, cellulitis, and Hoigne syndrome are the most important differential diagnoses.^3^ Generally, an immediate postinjection reaction in the injection site, along with ruling out other differential diagnoses, could lead to the diagnosis of NS.^8^ Furthermore, blood tests, electrocardiography (ECG), computerized tomography (CT) scans, sonography, and biopsy are among the diagnostic measures that help make a more reliable diagnosis.^3^, ^6^, ^9^, ^10^

Given that in this case, NS was diagnosed too late; the therapeutic measures were only symptomatic. Therefore, NS led to tissue necrosis, and the symptoms persisted for quite a long time. Early diagnosis and the adoption of prompt supportive therapeutic measures are fundamental for preventing and managing NS. Typically, systemic glucocorticoids and analgesics are prescribed for these patients.^8^, ^9^, ^11^ Anticoagulant agents (e.g., subcutaneous heparin or enoxaparin), blood diluents (e.g., oral pentoxifylline), or vasodilators (e.g., systemic alprostadil and transdermal nitroglycerine) may be used to accelerate the recovery process.^8^, ^9^, ^12^, ^13^ Furthermore, hyperbaric oxygen therapy has a role in accelerating the healing process.^14^

As mentioned in our patient, this complication did not occur in the buttock coadministered with betamethasone. Therefore, it is presumed that early administration of corticosteroids plays a preventive role in attenuating this complication. It seems that in addition to their therapeutic role, glucocorticoids can be used as an effective preventative measure in this syndrome. Although it is not suggested to consider corticosteroids as a general recommendation for anyone receiving such injections, because of the rarity of NS and adverse effects of corticosteroids, healthcare staff should be aware of the potential benefit of fast implementation of corticosteroids in cases diagnosed with NS.

There are several case reports of NS following the administration of benzathine penicillin. For example, an intramuscular injection of this drug in a 4‐year‐old child 8 h after the injection eventually resulted in amputation of the lower limb.^15^ Recently, two case reports of NS following intramuscular injection of benzathine penicillin have been reported as an ischemic necrotic event that occurs iatrogenically for the skin and deeper tissues.^16^ Therefore, it is necessary for physicians to consider this complication in parenteral injections, especially intramuscular injections, in cases of severe pain at the injection site.

More crucial than a quick diagnosis is the correct diagnosis of NS. Since there is no special diagnostic test for NS, the definite diagnosis requires ruling out other differential diagnoses and extensive laboratory and clinical evaluations. Furthermore, clinical consultations from other specialists are vital to ensure the true diagnosis fully. As a result, the absence of either of these parameters can lead to misdiagnosis or delayed diagnosis of this syndrome, which occurred in our patient. Moreover, in such cases, promptly initiating medical interventions is of the utmost importance.

Future studies should focus on the effectiveness of corticosteroids in different phases of NS, from the first minutes after injection to the following days or weeks after injection. Additionally, the development of diagnostic tools and techniques is needed to determine the etiology of NS vasculopathy, such as embolism, vasospasm, and thrombosis. As NS can lead to serious health issues and even mortality, any attempt toward the faster diagnosis of this syndrome would be worthwhile.

Although this case report opens a new window in the management of patients with NS, care must be taken in interpreting what has happened to the patient. In other words, in addition to the simultaneous employment of betamethasone, other therapeutic measures taken for the patient could have contributed to the patient's outcome, which we could not evaluate them retrospectively. Therefore, the beneficial effects seen in the patient may not be attributed solely to betamethasone. These other preventive measurements, such as the better‐homogenized penicillin suspension, a needle with proper length, and an appropriate injection technique such as the Z‐track technique, could have been applied in the right buttock that we were unable to evaluate them retrospectively (4).

## 4 CONCLUSION

Because Nicola syndrome is a rare complication of parenteral administration of drugs, especially intramuscularly, and there are no specific diagnostic tests for it, physicians should be suspected if severe stubbing pain at the injection site occurs. Based on this case, corticosteroids appear to play a protective role in preventing this complication.

## CONFLICTS OF INTEREST

All authors declare that they have no conflicts of interest to disclose.

## AUTHOR CONTRIBUTIONS

PM and BB contributed to the clinical data collection and prepared the case report. PM, BB, and MBO were involved in the diagnosis and treatment of the patient. PM and MBO contributed to the design of the case report presentation, responded to the reviewers’ comments, and performed the final revision of the manuscript. MBO supervised the project. All authors read and approved the final manuscript.

## ETHICAL APPROVAL

We hereby confirm that the present study conforms to the ethical standards and guidelines of the journal.

## CONSENT

Written informed consent was obtained from the patient for publication of this case report.

## Data Availability

The data that support the findings of this study are available from the corresponding author on reasonable request. The data are not publicly available due to privacy or ethics restrictions.
